# The beneficial effect of hydroxyapatite lasts: A randomized radiostereometric trial comparing hydroxyapatite-coated, uncoated, and cemented tibial components for up to 16 years

**DOI:** 10.3109/17453674.2012.665330

**Published:** 2012-04-24

**Authors:** Bart G Pijls, Edward R Valstar, Bart L Kaptein, Marta Fiocco, Rob GHH Nelissen

**Affiliations:** ^1^Bio Imaging Group (BIG), Department of Orthopaedics, Leiden University Medical Center, Leiden; ^2^Department of Biomechanical Engineering, Faculty 3mE, TU Delft; ^3^Department of Medical Statistics and Bioinformatics, Leiden University Medical Center, Leiden, the Netherlands

## Abstract

**Background and purpose:**

In contrast to early migration, the long-term migration of hydroxyapatite- (HA-) coated tibial components in TKA has been scantily reported. This randomized controlled trial investigated the long-term migration measured by radiostereometric analysis (RSA) of HA-coated, uncoated, and cemented tibial components in TKA.

**Patients and methods:**

68 knees were randomized to HA-coated (n = 24), uncoated (n = 20), and cemented (n = 24) components. All knees were prospectively followed for 11–16 years, or until death or revision. RSA was used to evaluate migration at yearly intervals. Clinical and radiographic evaluation was according to the Knee Society system. A generalized linear mixed model (GLMM, adjusted for age, sex, diagnosis, revisions, and BMI) was used to take into account the repeated-measurement design.

**Results:**

The present study involved 742 RSA analyses. The mean migration at 10 years was 1.66 mm for HA, 2.25 mm for uncoated and 0.79 mm for the cemented group (p < 0.001). The reduction of migration by HA as compared to uncoated components was most pronounced for subsidence and external rotation. 3 tibial components were revised for aseptic loosening (2 uncoated and 1 cemented), 3 for septic loosening (2 uncoated and 1 cemented), and 1 for instability (HA-coated). 2 of these cases were revised for secondary loosening after a period of stability: 1 case of osteolysis and 1 case of late infection. There were no statistically significant differences between the fixation groups regarding clinical or radiographic scores.

**Interpretation:**

HA reduces migration of uncemented tibial components. This beneficial effect lasts for more than 10 years. Cemented components showed the lowest migration. Longitudinal follow-up of TKA with RSA allows early detection of secondary loosening.

The early fixation properties of hydroxyapatite (HA) coatings on prostheses have been extensively studied (Geesink 2002). Animal studies have shown that HA may convert fibrous tissue into bone, and that even under unstable mechanical conditions HA is capable of inducing bone growth across peri-implant gaps (Soballe et al. 1992a,b, 1993). Additionally, radiostereometric (RSA) studies have shown reduced early migration of HA-coated tibial components compared to porous coated or non-coated tibial components in total knee arthroplasty (TKA) (Nelissen et al. 1998, Regner et al. 2000, Toksvig-Larsen et al. 2000, Therbo et al. 2008).

In contrast to early migration, the long-term migration of HA-coated tibial components has been scantily reported—as shown by a recent systematic review (Voigt and Mosier 2011). Thus, it is not clear whether the early biological fixation of HA-coated tibial components will endure and how the long-term migration compares to that of uncoated or cemented tibial components. Moreover, HA-specific complications such as delamination of the HA layer and third-body wear caused by HA particles have been reported in total hip arthroplasty and are potential problems in the long run (Bloebaum et al. 1994, Morscher et al. 1998).

We have already shown in a randomized radiostereometric trial of HA-coated, uncoated, and cemented tibial fixation that HA significantly reduces early migration compared to uncoated components (Nelissen et al. 1998). Here, we investigated the long-term (11- to 16-year) migration in these patients.

## Patients and methods

### Study design and patient demographics

68 consecutive posterior cruciate retaining TKAs (Interax; Howmedica, Rutherford, NJ) performed in 48 patients because of osteoarthritis or rheumatoid arthritis, were included in a randomized, controlled trial in an academic hospital between 1993 and 1998. The study was done in compliance with the Helsinki Declaration and was approved by the institutional ethics committee (pp 166/93; November 30, 1993), and patients gave informed consent. 24 TKAs were performed with cemented tibial components, 24 with HA-coated tibial components, and 20 with uncoated tibial components (Table 1).

**Table 1.** Baseline characteristics. Values are mean (SD)

	Cement	HA	Uncoated
	(n = 24)	(n = 24)	(n = 20)
Age	69 (8.6)	63 (11)	65 (15)
Sex (F:M)	18:6	21:3	16:4
Diagnosis (RA:OA:SA) **^a^**	15:9:0	17:6:1	17:3:0
BMI	26 (3.8)	27 (4.9)	24 (3.3)
Preoperative FTA angle	176 (8.1)	174 (8.7)	171 (12)
Preoperative KSS	27 (11)	22 (17)	25 (21)
Preoperative KSS (function)	14 (18)	14 (21)	5 (11)

**^a ^**RA: rheumatoid arthritis; OA: osteoarthritis; SA: sequelae after septic arthritis.

Reporting was in accordance with the CONSORT guidelines and the RSA guidelines (Moher et al. 2001,Valstar et al. 2005). 2-year migration results and details of patients and methods have been reported previously (Nelissen et al. 1998).

Fixation of the tibial component with HA was compared to uncoated fixation and to fixation with cement. The inserts were made of ultra-high-molecular-weight polyethylene (UHMWPE), sterilized by gamma radiation in air, and machined from ram-extruded GUR 415 resin containing calcium stearate.

**Figure 1.**
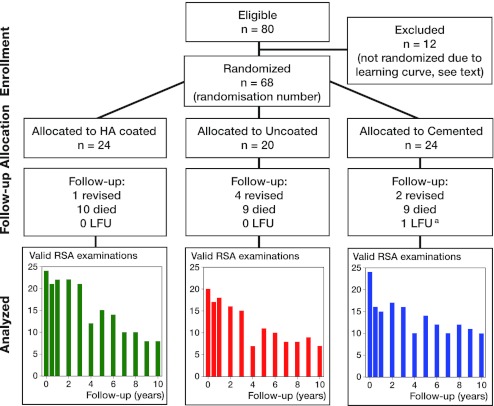
CONSORT flow chart.

In the present study, patients were followed for 11–16 years, or until death or revision of the tibial component (Figure 1). To account for the learning curve with this—at the time—new TKA at our institution (1992) and to gain experience with the RSA equipment, the first 12 TKAs were not randomized and received cemented fixation. These 12 TKAs were not included as part of the study. Nevertheless, RSA analysis was performed in order to exclude potential selection bias for the consecutive study. The migration was similar to that of the randomized cemented cases (p = 0.3), as analyzed with a generalized linear mixed model. During the study, patients remained blind regarding the fixation method. Observers were blinded during the RSA analysis regarding the presence or absence of HA coating, so the study was double-blind regarding the type of uncemented fixation (HA-coated or uncoated). Since cement is visible on (RSA) radiographs, the study was single-blind regarding the comparison with cement.

**^a ^**This patient moved out of the region at 3 years post-operatively, when the tibial component had stabilized at 0.64mm MTPM and there were no signs of loosening on the last radiograph.

### Surgical technique

All TKAs were performed by two experienced knee surgeons or under their direct supervision, and implanted through a standard midline incision and medial parapatellar arthrotomy. 6–8 tantalum markers were inserted into the tibial metaphysic bone before final implantation of the tibial component.

In the cemented group, Palacos bone cement (Schering, Kenilworth, NJ) was used after mechanical pulse-lavage of the cut bone surfaces. To allow migration measurements by marker-based RSA, three 2-mm Vitallium markers had been attached to the tibial component by the manufacturer.

### RSA technique

The RSA technique has been described previously (Nelissen et al. 1998). Analysis of the RSA examinations was performed with MBRSA 3.2 software (Medis Specials, Leiden, the Netherlands). The marker configuration model RSA technique was used for measurement of the pose of a rigid body in situations where less than 3 markers could be detected in both images of an RSA radiograph (Kaptein et al. 2005). In 2002, the calibration cage was replaced. Accuracy of the RSA set-up prior to 2002, as determined by double examination analysis (n = 40), was as follows for the translations expressed in means: x-axis 0.00 mm (SD 0.07 mm), y-axis 0.01 mm (SD 0.06 mm), and z-axis –0.02 mm (SD 0.13 mm) (Nelissen et al. 1998,Valstar et al. 2005). From 2002 onwards (n = 44), the accuracy was: x-axis 0.00 mm (SD 0.03 mm), y-axis 0.01 mm (SD 0.06 mm), and z-axis –0.01 mm (SD 0.08 mm) according to Kaptein et al. (2007). These values indicate a high level of precision for the measurement of migration of the tibial component relative to the bone and absence of any systematic bias.

Weight-bearing and flexion exercises were postponed until after the first RSA radiograph (1–5 days postoperatively). The patients were evaluated both clinically and by RSA examinations at predefined follow-up times (3 weeks, 6 weeks, 3 months, 6 months, and 1 year postoperatively) and then on an annual basis.

### Primary outcome: measurement of migration with RSA

The first RSA radiograph served as a baseline reference for the migration measurements.

Maximal total point motion (MTPM)—migration of the point on the prosthesis that has moved the most—was used to determine whether the groups were different regarding migration. When MTPM was different between the groups, translations and rotations along the x-, y-, and z-axis were evaluated to determine how they differed.

Most migration occurs in the first postoperative year, followed by either stabilization or continuous migration of the tibial components (Ryd et al. 1995). Since MTPM represents the length of a vector, which cannot be subjected to regular addition or subtraction, an additional RSA analysis was carried out with the 1-year postoperative RSA radiograph as a reference.

### Secondary outcome: clinical evaluation

Clinical evaluation was performed according to the Knee Society score (KSS) and Hospital for Special Surgery score (HSS) at each follow-up (Insall et al. 1989).

### Secondary outcome: radiographic evaluation

In addition to the RSA radiographs, conventional weight-bearing radiographs were acquired at 2-, 5-, 10-, and 15-year follow-up and graded according to the Knee Society roentgenographic evaluation: femoral-tibial aligment (FTA angle) and also alfa angle (frontal angle of the femoral component), beta angle (frontal angle of the tibial component), and delta angle (sagittal angle of the tibial component) (Ewald 1989).

### Statistics

Due to the high degree of accuracy of RSA, 20 TKAs were required for each trial arm—as was standard for RSA studies at the time the present study was designed (1992), (Albrektsson et al. 1992, Grewal et al. 1992). The results were analyzed according to the intention-to-treat principle. To take into account the repeated-measures design of the study, any missing follow-up occasion, variation in duration of follow-up, and bilaterality, and also to allow for confounder correction (Pijls et al. 2011), a generalized linear mixed model (GLMM) was used (R software version 2.12.0), which is considered to be the primary analysis method for this type of clinical study (DeSouza et al. 2009). In accordance with recent studies, a log-transformation was used for maximal total point motion (MTPM)—migration of the point on the prosthesis that has moved the most—because it is not normally distributed (Astephen Wilson et al. 2010). Due to multiple primary outcomes (translations, rotations), a Holm-Bonferroni correction for multiple testing was performed (Holm 1979). Means are presented until 10 years of follow-up. Afterwards, cases are presented individually. 95% confidence intervals (CIs) were calculated.

## Results

### Long-term migration

**Figure 2.**
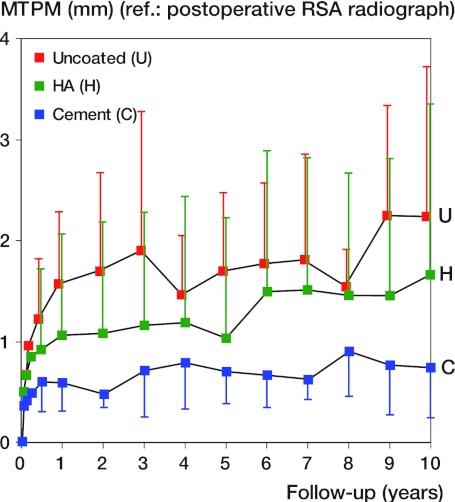
Migration in maximum total point motion (MTPM) (mean and standard deviation) according to the duration of follow-up in the hydroxyapatite (HA) group (green boxes), the uncoated group (red boxes), and the cemented group (blue boxes). The direct postoperative RSA radiograph is the reference. The groups differed significantly in migration (p < 0.001, GLMM). Missing values at 4-year follow-up were estimated as the mean of the 3-year and 5-year follow-up.

The migration analysis was composed of 742 RSA analyses using the direct postoperative RSA radiograph as reference. Figure 1 shows the number of valid RSA examinations for each follow-up occasion. Figure 2 shows the mean migration expressed in MTPM for each fixation group up to 10 years postoperatively. Throughout the follow-up period, the uncoated tibial components showed mean 0.39 mm (95% CI: 0.16–0.62) more migration than the HA-coated tibial components and mean 1.0 mm (CI: 0.82–1.18) more than the cemented tibial components, while the HA-coated components migrated mean 0.61 mm (CI: 0.42–0.80) more than the cemented components (unadjusted: p < 0.001, GLMM; and adjusted for age, sex, diagnosis, revisions, and BMI: p < 0.001, GLMM). The mean migration at 10 years was 1.61 mm for the osteoarthritis patients and 1.52 mm for the rheumatoid arthritis patients (p = 0.2, GLMM adjusted for fixation, age, sex, revision, and BMI).

To determine whether migration patterns varied between the groups, the mean translations and rotations were determined. The uncoated tibial components showed statistically significantly increased subsidence, external rotation, and lateral and anterior translation (in the order of clinical relevance). The addition of HA affected migration by decreasing subsidence by mean 0.26 mm (CI: 0.10–0.42) and external rotation by mean 0.47 degrees (CI: 0.27–0.67) compared to uncoated components.

**Figure 3.**
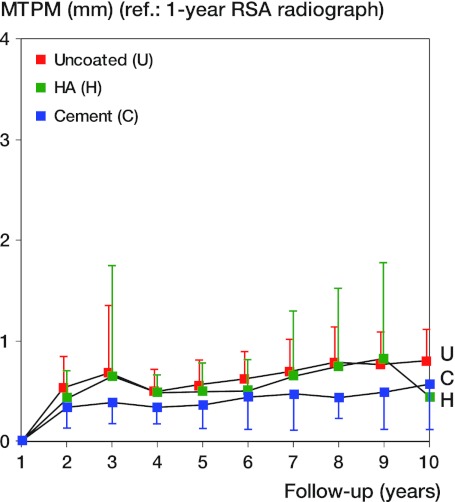
Migration from 1 to 10 years in maximum total point motion (MTPM) (mean and standard deviation) according to the duration of follow-up in the hydroxyapatite (HA) group (green boxes), the uncoated group (red boxes), and the cemented group (blue boxes). The 1-year postoperative RSA radiograph is the reference. The groups differed significantly in migration (p < 0.001, GLMM). Missing values at 4-year follow-up were estimated as the mean of the 3-year and 5-year follow-up.

463 RSA analyses composed the migration analysis relative to the first postoperative year (Figure 3). There was a statistically significant difference between the fixation groups regarding migration from 1 to 10 years (unadjusted: p < 0.001, GLMM; and p < 0.001, GLMM adjusted for age, sex, diagnosis, revisions, and BMI). After 1 year, the cemented tibial components migrated 0.043 mm/year, the HA-coated tibial components migrated 0.057mm/year, and the uncoated tibial components migrated 0.067 mm/year (p = 0.003, GLMM adjusted for age, sex, diagnosis, revisions, and BMI).

**Figure 4.**
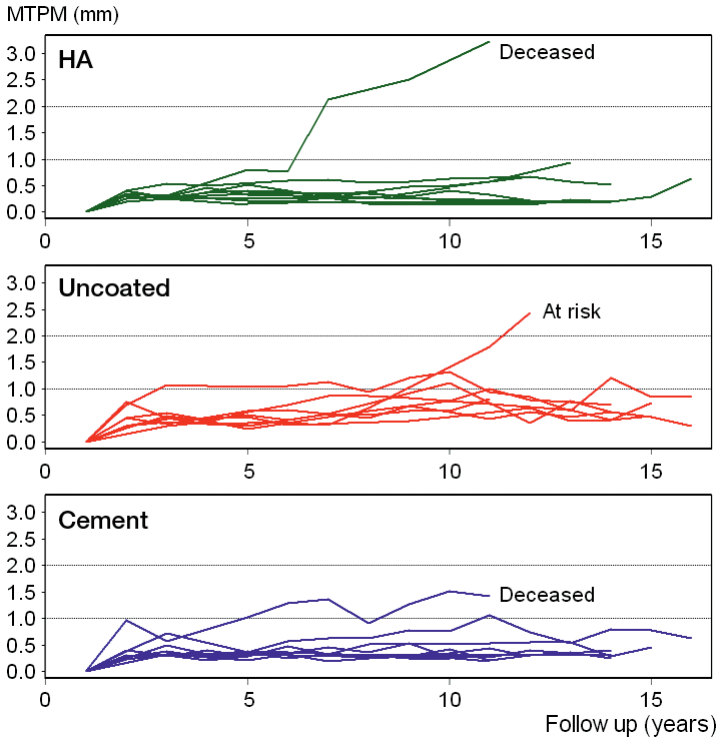
Migration from 1 to 16 years in maximum total point motion (MTPM) of individual cases with 10 years of RSA follow-up or more according to the duration of follow-up in the hydroxyapatite (HA) group (upper row), the uncoated group (middle row), and cemented group (lower row). The 1-year postoperative RSA radiograph was the reference.

Migration from 1 to 16 years for individual cases with 10 years or more of RSA follow-up is presented in Figure 4 according to fixation type: HA-coated (8 cases), uncoated (9 cases), and cemented (9 cases). There was 1 knee in the HA-coated group, 1 knee in the uncoated group and 1 knee in the cemented group with continuous migration. The patient in the HA-group and cemented group has died with the TKA in situ. The patient in the uncoated group is still alive and is considered to be at risk of aseptic loosening.

### Clinical evaluation

At 10 years postoperatively, there was a mean increase in KSS compared to preoperatively (59 (CI: 54–66)). There were no statistically significant or clinically relevant differences in KSS between the fixation types (p = 0.9, GLMM adjusted for age, sex, diagnosis, revisions, and BMI) (Table 2).

**Table 2.** Clinical and radiographic results

		Cement	HA	Uncoated
		Mean SD (95% CI)	Mean SD (95% CI)	Mean SD (95% CI)
Knee score **^a^**	5-year	81 11 (75–88)	81 13 (74–88)	83 5 (80–86)
	10-year	81 15 (71–91)	85 7 (80–90)	87 7 (81–92)
	Last FU **^f^**	76 18 (68–84)	86 10 (81–90)	81 14 (74–87)
Knee function score **^b^**	5-year	69 21 (55–82)	38 30 (20–55)	52 35 (30–75)
	10-year	45 29 (25–65)	46 33 (23–69)	42 32 (18–67)
	Last FU **^f^**	29 34 (14–44)	29 32 (15–43)	23 31 (8–38)
HSS **^b^**	5-year	48 8 (44–53)	46 11 (40–51)	49 5 (47–52)
	10-year	48 4 (45–50)	49 7 (44–53)	47 5 (43–51)
	Last FU **^f^**	41 17 (33–48)	51 14 (45–57)	41 14 (33–47)
Flexion **^d^**	5-year	109 16 (100–118)	100 15 (92–108)	101 14 (93–110)
	10-year	110 16 (100–120)	103 13 (93–113)	106 12 (95–110)
	Last FU **^f^**	105 13 (99–111)	106 12 (101–111)	101 17 (93–108)
FTA angle **^e^**	1-year	176 2.8 (175–177)	177 3.4 (176–178)	176 2.4 (175–177)
	5-year	177 2.3 (175–178)	177 4.1 (175–179)	177 2.9 (175 178)
	10-year	177 2.2 (175–178)	178 4.4 (175–181)	176 2.8 (174–178)
Alfa angle **^g^**	1-year	93 2.6 (92–95)	94 2.7 (93–95)	94 3.0 (93–96)
Beta angle **^h^**	1-year	90 1.7 (89–90)	88 3.0 (87–89)	89 2.0 (88–90)
Delta angle **^i^**	1-year	88 3.1 (86–89)	88 2.8 (87–89)	87 3.5 (86–89)

**^a^** p = 0.86, GLMM.

**^b^** p = 0.43, GLMM.

**^c^** p = 0.64, GLMM.

**^d^** p = 0.15, GLMM.

**^e^** p = 0.28, GLMM.

**^f^** Last FU at mean 9.0 years (range 3 months to 16 years) was calculated using the clinical score at the last available FU for each patient.

**^b^** HSS: Hospital for Special Surgery score

**^e^** FTA angle: femoral–tibial alignment

**^g ^**Alfa angle: frontal angle of the femoral component

**^h ^**Beta angle: frontal angle of the tibial component

**^i ^**Delta angle: sagittal angle of the tibial component.

At 10 years postoperatively, there was a mean increase in KSS function compared to preoperatively (33 (CI: 21–46)). There were no statistically significant or clinically relevant differences in KSS function between the fixation types (p = 0.4, GLMM adjusted for age, gender, diagnosis, revisions, and BMI) (Table 2). There were no significant differences in HSS or flexion between the fixation types.

### Radiographic evaluation

The FTA angles were similar between the fixation types (p = 0.3, GLMM adjusted for age, sex, diagnosis, revisions, and BMI) (Table 2). There were no statistically significant or clinically relevant differences in alfa, beta, or delta angles between the fixation types. Ten years postoperatively, there were 2 partial 2-mm radiolucent lines in the HA group, 1 partial 2-mm radiolucent line in the uncoated group, and no radiolucent lines of 2 mm or more in the cemented group.

### Revisions and exchanges of insert

**Figure 5.**
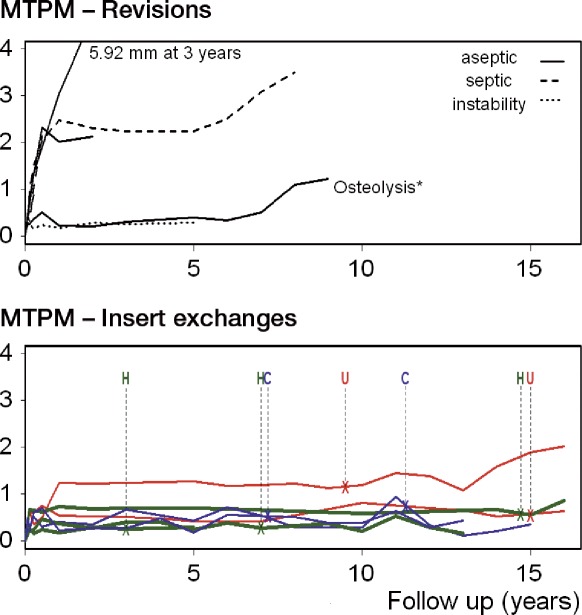
Individual migration patterns of the liner exchange and revised cases (with the postoperative radiograph as reference). For the insert exchanges, the letters at the top indicate the time of insert exchange with blue C for cemented tibial components, green H for HA-coated tibial components, and red U for uncoated tibial components. The tibial components remained securely fixed after the exchange of insert.

7 knees were revised: 3 tibial components for aseptic loosening (2 uncoated and 1 cemented), 3 for septic loosening (2 uncoated and 1 cemented), and 1 for instability (HA-coated). The individual migration patterns are shown in Figure 5. Of note is 1 case that was revised for secondary aseptic loosening after a period of stability. This secondary loosening was due to scalloping osteolysis at the tibial component located anterolaterally as identified on CT-scan and during the revision procedure. There was 1 case that was revised after late infection. This case also showed increasing migration after a stable period. There was 1 case of wound necrosis (cemented tibial component) early postoperatively that was treated successfully with surgical debridement and antibiotics, so the prosthesis was preserved.

* Secondary loosening due to osteolysis. Extensive antero-lateral osteolysis was seen on the CT-scan. This was confirmed at the revision procedure.

There were 7 PE insert exchanges for wear: 2 in the cemented group, 3 in the HA group, and 2 in the uncoated group. There was no statistically difference between the groups in the rate of insert exchange (with the numbers available; HA-coated vs. cemented, HR = 1.0, CI: 0.2–6.9; p = 1.0; and HA-coated vs. uncoated, HR = 0.6, CI: 0.1–5.6; p = 0.6).

## Discussion

We found different long-term migration between the 3 fixation types, with cemented components showing the lowest migration. For the uncemented components, HA reduces migration compared to the uncoated components and this clinically relevant effect endures beyond 10 years. The positive effect of HA was most noticeable in reducing subsidence and external rotation compared to the uncoated tibial components.

Negative effects of HA are the risk of HA delamination and third-body wear due to HA particles, as demonstrated in total hip arthroplasty (Bloebaum et al. 1994, Morscher et al. 1998). In the present study, the migration patterns of the HA-tibial components were stable at the long-term follow-up, so delamination of the HA coating was unlikely for the HA applied which was 60 μm thick and had a crystallinity of more than 90%. Crystallinity of more than 75% has been shown to provide adequate fixation and bone ingrowth (Overgaard et al. 1999). The rate of insert exchange in the HA group was comparable to that of the cemented and uncoated groups, thus no indication for accelerated third-body wear due to HA particles was anticipated. However, larger comparative studies are needed to fully address the potential influence of third-body wear by HA particles in TKA.

Early migration appears to predict long-term migration (Grewal et al. 1992, Ryd et al. 1995). Indeed, the increased (early) migration in the uncoated group compared to the cemented and HA group has been associated with an increased revision rate for the uncoated components. There were 2 cases of secondary loosening after a period of stability. Since these patterns have not been described before, there is a need for long-term RSA studies to further investigate these interesting migration patterns. The HA-coated components in our study also showed the well-described migration pattern for uncemented tibial components: substantial initial migration followed by stabilization (Nilsson et al. 2006, Henricson et al. 2008, Dunbar et al. 2009).

Compared to the HA-coated tibial components, the uncoated components showed more initial migration, which took more time to stabilize. Other RSA studies with follow-up ranging from 1 to 5 years have found similar results regarding the effect of HA on migration compared to porous coated and uncoated tibial components (Nelissen et al. 1998, Nilsson et al. 1999, 2006, Regner et al. 2000, Toksvig-Larsen et al. 2000, Carlsson et al. 2005, Therbo et al. 2008). In addition, recent clinical cohort series have illustrated that good long-term survival (with any reason for revision as endpoint) of 99% at 10 years and 98% at 10–15 years of follow-up can be achieved with similar HA-coated, posterior cruciate retaining tibial components (Cross and Parish 2005, Epinette and Manley 2007).

The magnitude of difference in migration from 1 to 10 years was less pronounced than the magnitude of difference in migration in the early postoperative period. It is not clear whether the differences in migration from 1 to 10 years are clinically relevant.

Knee Society scores and radiolographical outcome were similar in all groups. The high rate of insert exchange due to wear for the Interax TKA has been described in the literature and was judged to be caused by the type of sterilization of the polyethylene (gamma in air) and inappropriate shift of the load center on the tibial component, particularly in the smaller sized non-conforming inserts, causing excessive stress on the posteromedial and posterolateral surfaces (Sugimoto et al. 2005).

The strengths of our study are the long-term follow-up and the blinding for the presence or absence of the HA coating for both the patient and the observers. In surgical trials, blinding is often an issue (Devereaux et al. 2003). An HA coating, however, is ideal for a double-blind design, since it cannot be seen on radiographs, so the RSA analyzers and patients were blinded. On the other hand, cement is visible on (RSA) radiographs, so the study was only single-blind (patient) regarding the comparisons with cement. Nonetheless, migration analysis with RSA is a standardized and objective method with low susceptibility to different interpretations (Valstar et al. 2005). The risk of biased results for the cemented components is therefore negligible.

We should also note some limitations. Three-quarters of our patients suffered from end-stage rheumatoid arthritis. One could question whether the conclusions apply to osteoarthritis. However, the migration at 10 years was very similar between OA patients and RA patients (1.61 mm and 1.52 mm). The long-term migration of HA-coated tibial components compared to cemented components has been scantily reported (Voigt 2011). The early migration in our study is comparable to that found by Onsten et al. (1998), who included only OA patients. At 2 years, their cemented components migrated (MTPM) approximately 0.6 mm and their HA components migrated approximately 1.0 mm. These migrations are similar to our results; OA or RA did not influence the effect of HA on long-term migration of the tibial components.

In conclusion, HA reduces migration of uncemented tibial components, which was most pronounced in the first postoperative years. The beneficial effect of HA endures beyond 10 years and there is no evidence for delamination of the HA layer. Since cemented components showed the lowest migration throughout the follow-up and have excellent survival in the registries, cement is a safe choice for fixation of the tibial component. Longitudinal follow-up of TKA with RSA allows early detection of secondary loosening.
